# Fungal Methane Production Under High Hydrostatic Pressure in Deep Subseafloor Sediments

**DOI:** 10.3390/microorganisms12112160

**Published:** 2024-10-26

**Authors:** Mengshi Zhao, Dongxu Li, Jie Liu, Jiasong Fang, Changhong Liu

**Affiliations:** 1 State Key Laboratory of Pharmaceutical Biotechnology, Nanjing University, Nanjing 210023, China; 602022300055@smail.nju.edu.cn (M.Z.); laolang_2012@163.com (D.L.); 2 Shanghai Engineering Research Center of Hadal Science and Technology, College of Marine Sciences, Shanghai Ocean University, Shanghai 201306, China; d220200055@st.shou.edu.cn; 3 Laboratory for Marine Biology and Biotechnology, Qingdao Marine Science and Technology Center, Qingdao 266071, China

**Keywords:** anaerobic, HHP, *Schizophyllum commune* 20R-7-F01, CH_4_, transcriptomics, ROS

## Abstract

Fungi inhabiting deep subseafloor sediments have been shown to possess anaerobic methane (CH_4_) production capabilities under atmospheric conditions. However, their ability to produce CH_4_ under in situ conditions with high hydrostatic pressure (HHP) remains unclear. Here, *Schizophyllum commune* 20R-7-F01, isolated from ~2 km below the seafloor, was cultured in Seawater Medium (SM) in culture bottles fitted with sterile syringes for pressure equilibration. Subsequently, these culture bottles were transferred into 1 L stainless steel pressure vessels at 30 °C for 5 days to simulate in situ HHP and anaerobic environments. Our comprehensive analysis of bioactivity, biomass, and transcriptomics revealed that the *S. commune* not only survived but significantly enhanced CH_4_ production, reaching approximately 2.5 times higher levels under 35 MPa HHP compared to 0.1 MPa standard atmospheric pressure. Pathways associated with carbohydrate metabolism, methylation, hydrolase activity, cysteine and methionine metabolism, and oxidoreductase activity were notably activated under HHP. Specifically, key genes involved in fungal anaerobic CH_4_ synthesis, including methyltransferase mct1 and dehalogenase dh3, were upregulated 7.9- and 12.5-fold, respectively, under HHP. Enhanced CH_4_ production under HHP was primarily attributed to oxidative stress induced by pressure, supported by intracellular reactive oxygen species (ROS) levels and comparative treatments with cadmium chloride and hydrogen peroxide. These results may provide a strong theoretical basis and practical guidance for future studies on the contribution of fungi to global CH_4_ flux.

## 1. Introduction

Methane (CH_4_) is a potent greenhouse gas pivotal to global climate dynamics, primarily sourced from biogenic emissions driven by microbial activity, which constitute approximately 90% of the global CH_4_ budget, estimated at 380–755 Tg annually. Significant contributors include coal beds, seafloor sediments, and subsurface reservoirs, with deep-sea sediments alone contributing around 20% of these emissions [1,2,3].

Archaea are well documented as major producers of CH_4_, utilizing various biochemical pathways, such as CO_2_ reduction with H_2_, acetate reduction, and methylotrophic pathways involving methanol and methoxy-group-containing substrates [4]. Recent research has also identified aerobic bacterial pathways contributing to CH_4_ production, including methylthio-alkane reductase’s involvement in methionine biosynthesis and C-P lyase in phosphonate ester degradation [5,6]. Furthermore, both plant and animal cells have been observed releasing CH_4_ under aerobic conditions independently of endosymbionts, although the precise mechanisms remain elusive [7,8]. In contrast, fungi, a vital group of eukaryotic organisms, have received less attention concerning CH_4_ production. Lenhart et al. (2012) provided initial evidence that wood-decaying fungi such as *Pleurotus sapidus*, *Trametes versicolor*, *Lentinula edodes*, *Laetiporus sulphureus*, and *Hypholoma fasciculare* produce CH_4_ under aerobic conditions, identifying serine as a precursor for methane synthesis [9]. Subsequent studies by Ernst et al. (2022) presented contradictory findings, showing that fungi like *Saccharomyces cerevisiae* S288C and *Aspergillus niger* DSM 821 primarily generate CH_4_ via Fenton chemistry rather than enzymatic reactions [10].

Recent research by Huang et al. (2022), employing biochemical, genetic, and stable isotopic tracer analyses, revealed that strains of *Schizophyllum commune* 20R-7-F01, isolated from coal-bearing sediments ~2 km below the seafloor (under 35 MPa hydrostatic pressure), utilized a novel halomethane-dependent pathway for CH_4_ production during anaerobic degradation of phenol, benzoic acid mono- and polymers, and cyclic sugars [11]. The taxonomic classification of *S. commune* is complicated by its widespread distribution and genetic diversity. Traditionally viewed as a single species, recent genomic analyses reveal significant variation among strains. Notably, the subseafloor *S. commune* 20R-7-F01 shows genetic divergence from terrestrial strains like *S. commune* H4-8, with many genes lacking orthologs [12]. Similar anaerobic methanogenic pathways have been confirmed in other wood-rot fungi, such as *Agaricus bisporus*, *Hypsizygus marmoreus*, and *Pleurotus ostreatus* [13,14,15]. Nonetheless, uncertainties persist regarding the ability of these fungi to produce CH_4_ under in situ high hydrostatic pressure (HHP) conditions, as well as specific mechanism governing CH_4_ production in response to HHP.

In this study, we cultured the fungal strain *S. commune* 20R-7-F01 anaerobically in 1 L stainless steel vessels at 30 °C to mimic in situ subseafloor environments. Our findings revealed a significant increase in CH_4_ production by this subseafloor fungus under elevated hydrostatic pressure (HP), primarily attributed to the induction of reactive oxygen species (ROS) by HHP. These results likely highlight the potential role of fungi as CH_4_ producers in the deep biosphere, an aspect that may have been previously underestimated in global CH_4_ budgets.

## 2. Materials and Methods

### 2.1. High Hydrostatic Pressure Cultivation Experiments

*Schizophyllum commune* 20R-7-F01 (CGMCC 11604) was isolated from a sediment core collected at a depth of 1966 m below seafloor (mbsf) from the Western Pacific Ocean [16]. Mycelial inocula were prepared following the method described by Zain Ul Arifeen et al. (2021). For the high hydrostatic pressure (HHP) cultivation experiments, fresh mycelial inocula (7 g) were introduced into 170 mL sterile culture bottles containing Seawater Medium (SM). The SM composition included CaCl_2_ (2.99 g/L), MgCl_2_ (4.17 g/L), KBr (0.10 g/L), NH_4_Cl (0.16 g/L), KCl (5.05 g/L), NaCl (33.43 g/L), H_3_BO_3_(0.02 g/L), Na_2_SO_4_ (0.21 g/L), and C_6_H_12_O_6_ (20 g/L). The bottles were then purged with 99.99% N_2_ for 15 min to remove oxygen from the culture bottles [17]. Culture bottles, fitted with sterile syringes for pressure equilibration, were incubated at 30 °C under HP of 15 MPa and 35 MPa, achieved by manual pumping of water into the vessel(TOP INDUSTRIE, Paris, France). A control culture under standard atmospheric pressure (0.1 MPa) was maintained under identical conditions. Fungal mycelia were harvested after 1, 3, and 5 days of culture, and one vial of mycelia was filtered through sterile gauze, rinsed three times with deionized water, immediately treated with liquid nitrogen, and stored at −80 °C for transcriptome analysis. Additionally, three replicates of harvested mycelia underwent the same gauze filtration and rinsing steps before being immediately utilized for biomass and CH_4_ quantification. Simultaneously, one vial of mycelia was also employed for assessing cellular activity following the aforementioned procedures.

For the ROS testing experiments, fresh mycelial inocula (7 g) were inoculated into 170 mL culture bottles containing SM supplemented with 0.75 mM, 1.5 mM, and 3 mM concentrations of cadmium chloride (Sigma-Aldrich, Shanghai, China) or hydrogen peroxide (Sigma-Aldrich, Shanghai, China) [18,19]. Culture bottles were incubated at 30 °C under standard atmospheric pressure. Fungal mycelia were harvested after 1, 3, and 5 days of incubation. Subsequently, three replicates of harvested mycelia underwent the same gauze filtration and rinsing steps before being immediately utilized for biomass and CH_4_ quantification. Simultaneously, one vial of mycelia was also employed for assessing cellular activity following the aforementioned procedures.

### 2.2. Assessment of Fungal Hyphal Vitality and Biomass Determination

To assess fungal mycelial viability, a 0.4% trypan blue (Sigma-Aldrich, Shanghai, China) staining technique was employed [20]. Viable mycelial cells were identified as colorless under optical microscopy (XIUILAB, Shanghai, China), whereas non-viable cells were stained blue. Mycelia harvested by filtration were dried in a 65 °C oven for one day to determine biomass. Dry weights were measured to quantify biomass production [21].

### 2.3. Assessment of ROS, O^2−^, OH·, H_2_O_2_, and CH_4_ Levels in Fungal Mycelia

Intracellular levels of ROS in fungal mycelia were assessed following established methodologies [22,23]. The 2′,7′-dichlorofluorescein diacetate (DCFH-DA, Beyotime, Shanghai, China) probe was introduced into cells, and ROS concentrations were determined through fluorescence microscopy examination and subsequent quantification using ImageJ software (version 1.55i). The intracellular content of O^2−^ in fungal mycelia was determined as per established protocols [24,25]. The superoxide anion reacts with hydroxylamine hydrochloride to form NO^2−^, which, upon reaction with p-aminobenzenesulfonami-de and naphthalene ethylenediamine hydrochloride, produces a red azo compound with a characteristic absorption peak at 530 nm. The content of O^2−^ can be determined based on the absorbance at 530 nm. The intracellular content of OH· in fungal mycelia was determined as per established protocols [26]. The hydroxyphenyl fluorescein (HPF,) probe was introduced into cells, and OH· concentrations were determined through fluorescence microscopy examination and subsequent quantification using ImageJ software (version 1.55i). The intracellular content of H_2_O_2_ in fungal mycelia was determined as per established protocols [27,28]. Absorbance at 415 nm, resulting from the formation of a titanium peroxide complex (Ti^4+^ and H_2_O_2_), was measured to quantify H_2_O_2_ concentrations. Intracellular CH_4_ levels in fungal mycelia were evaluated following detailed procedures, utilizing gas chromatography (GC, HP-Agilent, Shanghai, China) for accurate quantification of CH_4_.

### 2.4. Transcriptomic Analysis

Mycelial samples for transcriptome sequencing were labeled as follows: “d1_01M”, “d3_01M”, “d5_01M”, “d1_15M”, “d3_15M”, “d5_15M”, “d1_35M”, “d3_35M”, and “d5_35M”. Here, “dn” denotes sampling days (1, 3, and 5 days), and “nM” indicates pressure levels (0.1, 15, and 35 MPa) applied to the strain. Total RNA extraction utilized TRIzol reagent (TIANGEN, Beijing, China) per the manufacturer’s instructions, followed by cDNA library construction. Sequencing employed the Illumina HiSeq platform with default RNA protocols (Meiji, Shanghai, China). Clean reads were obtained by removing adapters, sequences with >10% N bases, and low-quality sequences (Phred score Q ≤ 5, >50% of reads) (Table S1). Clean reads were mapped to the strain 20R-7-F01 assembled genome (BioProject ID: PRJNA544166) using TopHat2 [29]. Gene expression levels were quantified in Transcripts Per Million (TPM) using Cufflinks software (version 2.2.1) [30]. Raw RNA-seq data were deposited in the NCBI Sequence Read Archive under BioProject ID PRJNA1101667.

Differential gene expression analyses utilized the DESeq method DESeq2 [31], applying a threshold of *p*-value < 0.05 and |Log2 (fold-change)| ≥ 1 to identify significant DEGs [32]. Functional analysis of DEGs included gene ontology (GO) and Kyoto Encyclopedia of Genes and Genomes (KEGG) enrichment analyses using clusterProfiler in R (v4.3.0). Enriched pathways were visualized with the Pathview package, setting the threshold for enriched gene annotations at *p*-value < 0.05. DEGs related to key pathways underwent hierarchical clustering, with correlation analysis performed using psych and reshape 2 packages in R (v4.3.0). A correlation network of pathway genes was constructed using Cytoscape software version 3.9.1 (https://cytoscape.org/releasenotes.html, accessed on 12 May 2024).

### 2.5. Quantitative Real-Time PCR Analysis

Quantitative real-time PCR (qRT-PCR) followed the protocol outlined by Zain Ul Arifeen et al. Fungal cultures were maintained under identical conditions and durations to those for RNA-seq samples. SYBR qPCR Master Mix (Vazyme, Nanjing, China) and specific primer pairs for each gene (Table S2) were used for qRT-PCR analysis. Thermal cycling conditions included initial denaturation at 95 °C for 30 s, followed by 43 cycles of 95 °C for 10 s, 58.5 °C for 30 s, and 72 °C for 30 s. Relative gene expression was calculated using the 2^−ΔΔCT^ method [33].

### 2.6. Statistical Analysis

The data were presented as mean ± standard deviation. One-way analysis of variance (ANOVA) or Student’s *t*-test, performed using GraphPad Prism version 8.0.2, was used to analyze significant differences between treatments (*p* < 0.05).

## 3. Results and Discussion

### 3.1. Impact of High Hydrostatic Pressure on Fungal Methane Productivity

**Figure 1:**
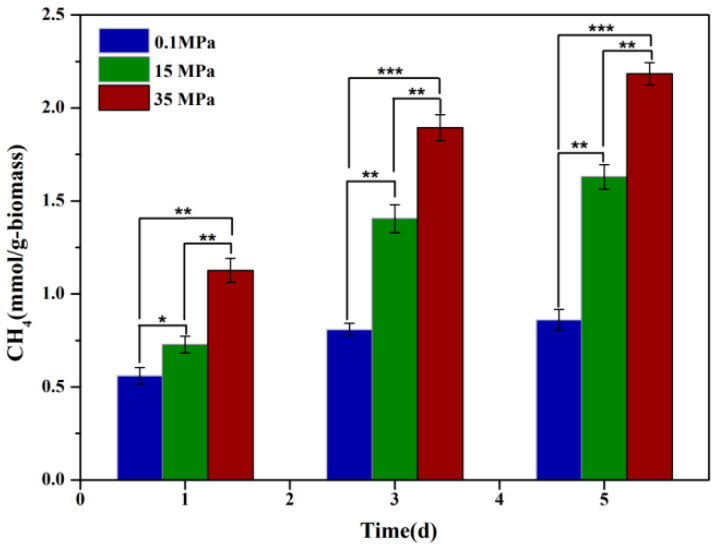
Methane production by strain 20R-7-F01 under varying hydrostatic pressures. Note: * represents *p* < 0.05; ** represents *p* < 0.01; *** represents *p* < 0.001.

To assess the effect of HHP on fungal CH_4_ productivity, strain 20R-7-F01 was cultured in bottles under varying HHP conditions, and the CH_4_ yield in the headspace was quantified using GC. The results demonstrated a significant enhancement in the CH_4_ production of strain 20R-7-F01 with increasing HP(Figure 1). Specifically, CH_4_ production at 15 MPa was approximately 1.3, 1.7, and 1.9 times higher on days 1, 3, and 5 of culture, respectively, compared to atmospheric conditions. Furthermore, at 35 MPa (equivalent to in situ pressure), CH_4_ production increased to approximately 2.0, 2.4, and 2.5 times higher than at atmospheric pressure. This substantial increase suggests a strong influence of HHP in enhancing CH_4_ production by strain 20-7-1. The effect of hydrostatic pressure on biological methanogenesis may be a universal phenomenon that affects all methanogens, but similar observations have only been noted in archaea, such as *Thermophilic marburgensis*, which exhibited CH_4_ production levels approximately 3 times higher than atmospheric levels when cultured at 50 MPa [34]. The mechanism behind enhanced CH_4_ production under HHP in archaea is hypothesized to involve oxidative stress induced by HP. However, it remains unclear whether analogous mechanisms govern the impact of HHP on fungal CH_4_ production.

### 3.2. Transcriptomic Analysis of Methane Synthesis Genes Under High Hydrostatic Pressure

The transcriptomic analysis revealed significant upregulation of key genes involved in methane synthesis under high hydrostatic pressure conditions in *S. commune* 20R-7-F01. Following cultivation at 15 MPa for 3 days, the expression levels of *mct1*, *dh3*, and *ms* increased by 5.9-fold, 2.8-fold, and 2.7-fold, respectively, compared to ambient pressure (0.1 MPa) (Table 1). Similarly, at 35 MPa, these genes showed increases of 7.9-fold, 4.5-fold, and 3.1-fold, respectively (Table 1). The quantitative PCR validated these findings, demonstrating significant upregulation of *mct1*, *dh3*, and *ms* under HHP conditions (Table 1). This enhanced methane production is primarily attributed to the upregulation of genes encoding key enzymes involved in methane synthesis.

**Figure 2:**
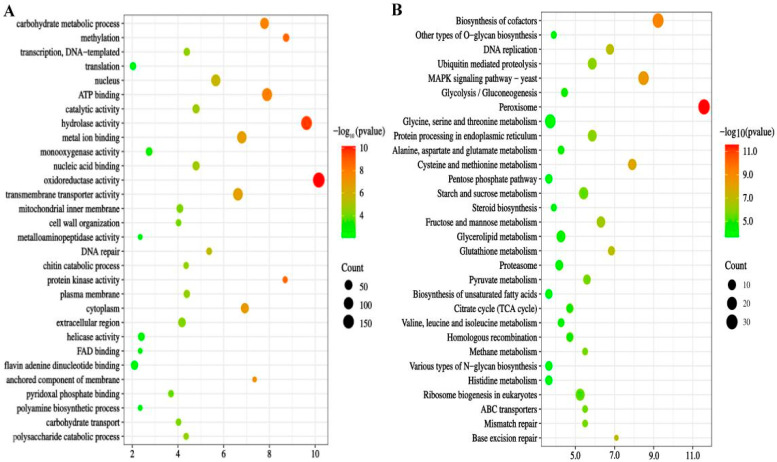
Enrichment analysis of significantly differentially expressed genes (DEGs). Panel (**A**) depicts GO enrichment analyses of the 2316 DEGs. Panel (**B**) shows KEGG enrichment analyses of the 2316 DEGs. Description of the top 30 enriched GO and KEGG pathways.

The transcriptional correlation analysis identified 2316 differentially expressed genes (DEGs) highly correlated (|*p*| ≥ 0.9) with mct1 (689 DEGs), dh3 (817 DEGs), and ms (810 DEGs) (Table S1). The gene ontology (GO) enrichment analysis revealed that these DEGs were enriched in activities associated with oxidoreductase functions, carbohydrate metabolism, methylation, ATP binding, hydrolase activity, metal ion binding, and transmembrane transport. Notably, oxidoreductase activity exhibited the highest enrichment, comprising 8.5% of the total 2316 DEGs (*p* = 6.95 × 10^^−11^) (Figure 2A, Table S1). The KEGG pathway enrichment analysis further indicated significant enrichment of these DEGs in pathways such as peroxisomes, glycolysis/gluconeogenesis, the tricarboxylic acid cycle, and the pentose phosphate pathway under HHP conditions (Figure 2B).

**Figure 3:**
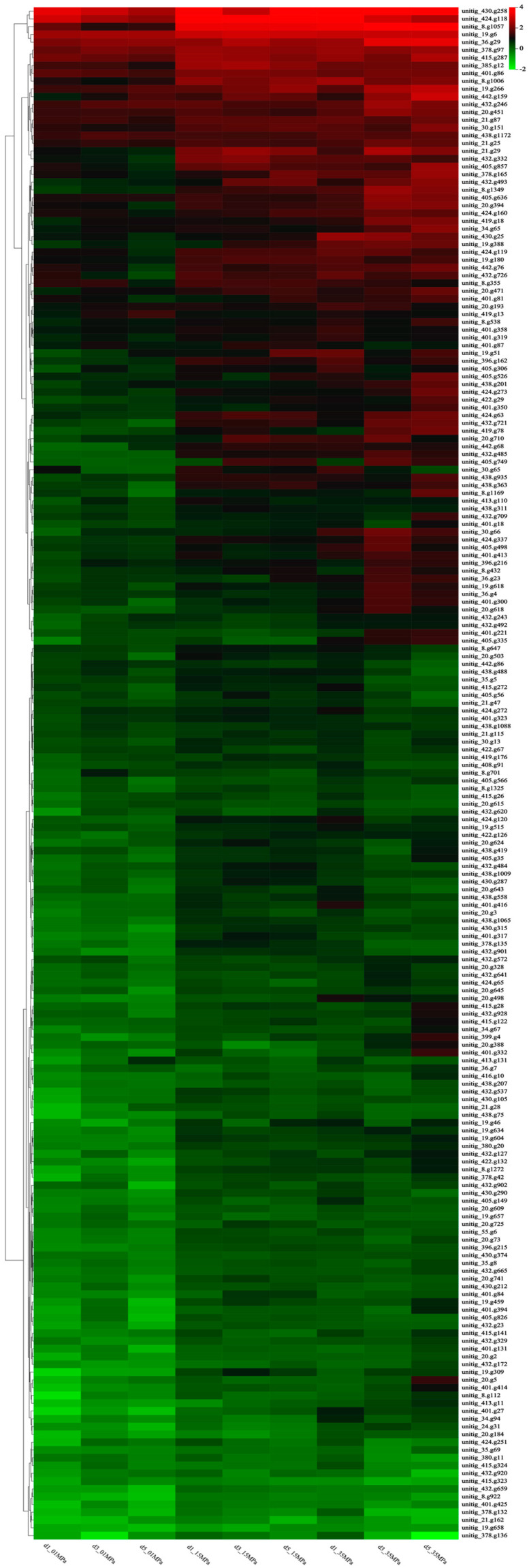
Hierarchical clustering heat map analysis of DEGs in the oxidoreductase activity pathway.

The hierarchical clustering analysis illustrated the upregulation of genes involved in oxidoreductase activities, particularly those implicated in oxidative stress response (e.g., *SOD*, *CAT*, and *BCP*), in *S. commune* 20R-7-F01 under HHP conditions (Figure 3, Table S2). Collectively, these findings suggest that the enhancement of methane synthesis metabolism under HHP conditions may be linked to alterations in oxidoreductase activities. Similar observations in other piezophilic organisms, such as *Sporosarcina psychrophila* DSM 6497 and *Shewanella piezotolerans* WP3, underscore that *S. commune* 20R-7-F01, like other piezophiles, counters oxidative stress induced by HHP through the activation of oxidative–reductive pathways [35,36].

**Figure 4:**
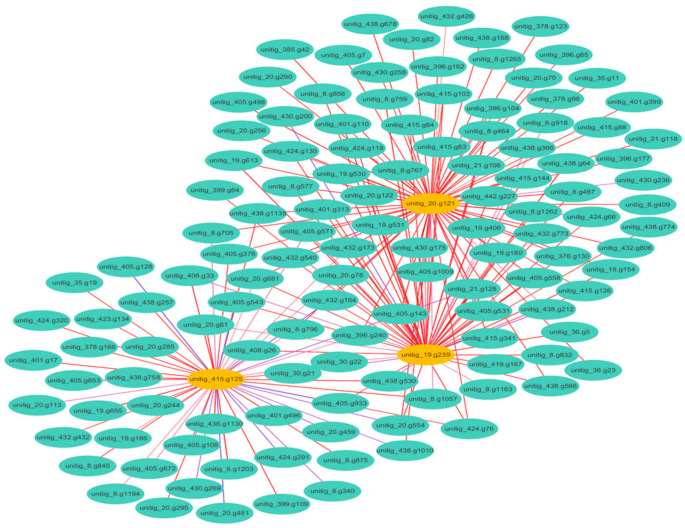
A network diagram illustrating the interactions between DEGs associated with methane production and oxidative stress. Each node represents a gene, while edges represent interactions.

Furthermore, the investigation of DEGs related to antioxidant genes revealed significant correlations with key genes involved in methane biosynthesis in strain 20R-7-F01 (Figure 4). A total of 197 DEGs of antioxidant genes were identified, with 54 (27.4%) showing notable correlations with *mct1*, 79 (40.1%) with *dh3*, and 64 (32.5%) with *metE*. These findings indicate that these antioxidant genes were significantly involved in methane production by *S. commune* under HHP conditions. Enhanced methane release in response to oxidative stress induced by HHP may serve as a protective mechanism against biological membrane damage caused by reactive oxygen species (ROS) [37,38]. In summary, our study highlights that the increased methane production observed in *S. commune* 20R-7-F01 under HHP conditions is a response to oxidative stress induced by HHP, mediated through the upregulation of genes associated with both methane synthesis and antioxidant defense mechanisms. These findings contribute to our understanding of microbial adaptation to extreme environmental conditions, emphasizing the role of methane production in stress response strategies.

### 3.3. ROS and H_2_O_2_ Induced in Fungal Cells by High Hydrostatic Pressure

To explore the enhanced methane production of *S. commune* 20R-7-F01 under high hydrostatic pressure (HHP) as a potential response to elevated fungal cell ROS and H_2_O_2_ levels induced by HHP, we assessed intracellular ROS and H_2_O_2_ levels, as well as the activities of antioxidative enzymes (*SOD*, *CAT*, *POD*) at 0.1 (control), 15, and 35 MPa pressures. Our results revealed significant increases in ROS and H_2_O_2_ levels in fungal cells under HHP compared to 0.1 MPa, accompanied by reduced fungal cell viability (Table 2, Figure S1). For instance, after 5 days of cultivation, the ROS and H_2_O_2_ levels were 7.04-fold and 6.12-fold higher at 15 MPa, and 10.33-fold and 8.51-fold higher at 35 MPa, respectively, compared to at 0.1 MPa, yet the cells were clearly labeled blue, with trypan blue. Further analysis demonstrated that the increase in ROS levels induced by HHP was primarily due to changes in H_2_O_2_ levels, while the contributions of superoxide anion (O^2−^) and hydroxyl radical (OH·) were relatively insignificant (Table S3). This is similar to findings by Zhe et al. (2018), who reported oxidative damage in the deep-sea bacterium *Shewanella piezotolerans* WP3 due to elevated intracellular H_2_O_2_ levels under 20 MPa conditions [39]. Additionally, the antioxidative enzyme activities within the fungal hyphae increased with pressure, notably with peroxidase (*POD*) activity at 35 MPa after 5 days of cultivation, which was 5.95 times higher than at 0.1 MPa over the same period. This is consistent with the results shown in Figure 3. The heightened activities of *SOD*, *CAT*, and *POD* in scavenging ROS- and H_2_O_2_-induced cellular damage under HHP suggest that *S. commune*, in response to HHP stress, employed metabolic mechanisms akin to those observed in the deep-sea bacterium *Shewanella piezotolerans* WP3 and yeast *Saccharomyces cerevisiae* [40,41].

**Figure 5:**
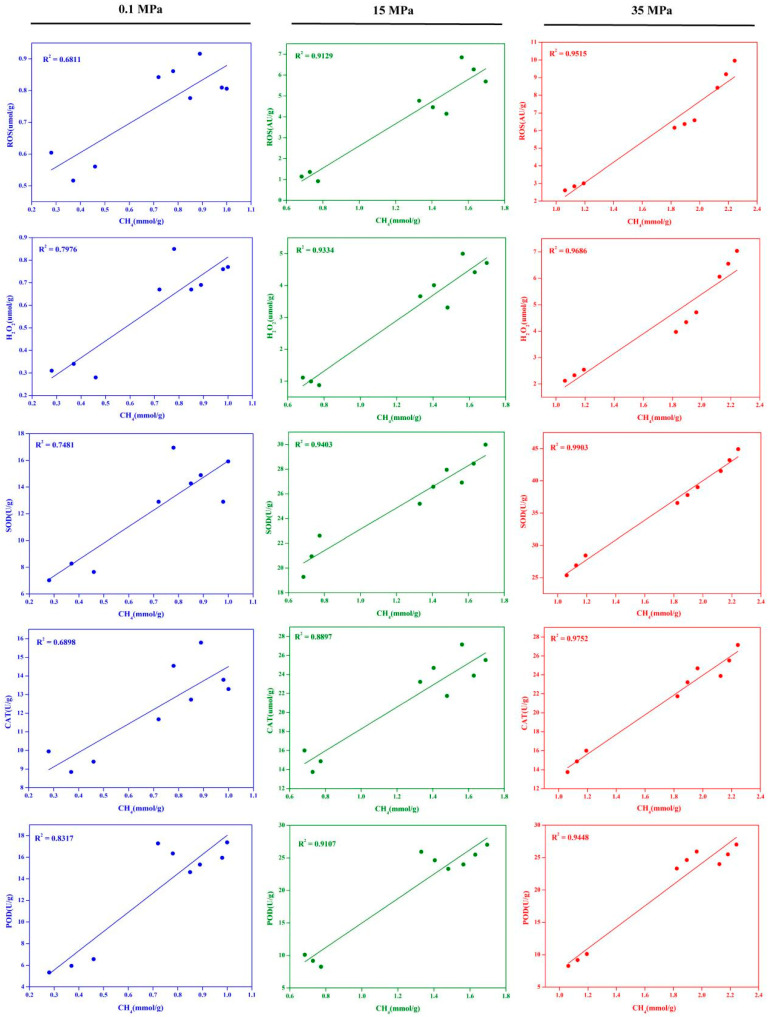
The correlation between methane production and the activities of ROS, H_2_O_2_, SOD, CAT, and POD in *S. commune* 20R-7-F01.

After establishing the occurrence of oxidative damage in *S. commune* 20R-7-F01 under HHP conditions, we conducted a correlation analysis between intracellular ROS and H_2_O_2_ levels, antioxidant enzyme activities (*SOD*, *CAT*, and *POD*), and methane content across different pressures. The results depicted in Figure 5 reveal that at 0.1 MPa, there existed a moderate positive correlation between methane production in the strain and intracellular ROS and H_2_O_2_ levels, as well as antioxidant enzyme activities, although this was not statistically significant. In contrast, at 15 MPa and 35 MPa, significant positive correlations were observed between methane production and intracellular ROS and H_2_O_2_ levels, as well as antioxidant enzyme activities. Particularly notable was the pronounced positive correlation between methane production and H_2_O_2_ levels. For instance, at 15 MPa, the correlation coefficient between methane production and H_2_O_2_ levels in the strain reached 0.9334. Similarly, at 35 MPa, the correlation coefficient between methane production and H_2_O_2_ levels also reached 0.9686. These results indicate that alongside the increase in intracellular H_2_O_2_ levels in *S. commune* 20R-7-F01, there is a corresponding increase in methane release. This further corroborates the conclusion from our transcriptome analysis showing that enhancing methane metabolism in *S. commune* 20R-7-F01 is a fungal response mechanism to HHP-induced ROS. While Ernst et al. (2022) [10] have demonstrated the existence of an ROS-driven methane production mechanism in general organisms, this is based on Fenton chemistry rather than a direct ROS-driven pathway specific to methane production in organisms. Similarly, although Mauerhofer et al. (2021) [34] found an increase in methane production by methanogenic archaea with increasing HHP, they did not identify the fundamental reasons behind the HHP-induced enhancement of methane production in archaea [33]. Here, we provide the first evidence that HHP can promote methane production in *S. commune* 20R-7-F01 for adapting to oxidative stress.

### 3.4. Experimental Evidence of ROS Contribution to Increased Methane Production in S. commune 20R-7-F01

To investigate the influence of oxidative stress induced by H_2_O_2_ or CdCl_2_ on methane production by *S. commune* 20R-7-F01, we cultured the fungus in a liquid mPD medium supplemented with varying concentrations (0.75 mM, 1.5 mM, and 3 mM) of these stressors. Control cultures lacking H_2_O_2_ or CdCl_2_ served as a baseline. Methane production capacity was assessed under atmospheric pressure conditions after 1, 3, and 5 days of cultivation. As detailed in Table 3, the methane production markedly increased with increasing concentrations of H_2_O_2_ or CdCl_2_. For instance, exposure to 0.75 mM CdCl_2_ resulted in methane production 1.45 times higher than the control after five days, which further increased to 2.44 times higher with 3 mM CdCl_2_. Similarly, exposure to 0.75 mM H_2_O_2_ led to a 1.74-fold enhancement in methane production relative to the control condition, while increasing the H_2_O_2_ concentration to 3 mM further augmented methane production by a factor of 3.06. It is noteworthy that supplementation of the mPD culture medium with BHT to scavenge intracellular ROS under different oxidative stress conditions led to a corresponding decrease in methane production by the strain. For example, the addition of antioxidant BHT (5 mM) to the fungal mycelium exposed to 3 mM H_2_O_2_ for five days resulted in a significant reduction in methane production from 2.63 mmol/g to 2.23 mmol/g. Concurrently, increasing concentrations of H_2_O_2_ or CdCl_2_ were correlated with elevated levels of intracellular ROS (Table S4), accompanied by reduced fungal cell viability and biomass (Figures S2 and S3). For instance, exposure to 3 mM H_2_O_2_ led to a decrease in biomass by 45.27 mg relative to the control, accompanied by significant trypan blue staining of the cells. These findings collectively underscore that oxidative stress induced by H_2_O_2_ or CdCl_2_ promotes methane production by *S. commune* 20R-7-F01. Thus, oxidative damage induced by HHP likely contributes to the increased methane production by this fungus. Similar observations by Gu et al. and Samma et al. in alfalfa roots indicate that elevated ROS levels from metal stressors enhance methane emission [17,18].

## 4. Conclusions

In conclusion, our study demonstrates that *Schizophyllum commune* 20R-7-F01, isolated from the subseafloor sediment approximately 2 km below the seabed, exhibited enhanced methane (CH_4_) production capabilities under in situ temperature, high hydrostatic pressure, and anaerobic conditions. Through comprehensive analyses encompassing biological activity assays, biomass quantification, transcriptomics, and metabolomics, we found that *S. commune* not only survived but significantly increased CH_4_ production under HHP conditions. Pathways related to carbohydrate metabolism, methylation, hydrolase activity, and the metabolism of cysteine and methionine, as well as activities of redox enzymes, were notably activated under HHP. Specifically, critical genes involved in fungal anaerobic CH_4_ synthesis, such as methyltransferase *mct1* and dehalogenase *dh3*, were markedly upregulated. The observed enhancement in CH_4_ production under HHP was primarily attributed to pressure-induced oxidative stress, supported by comparative analyses of intracellular ROS levels and treatments involving cadmium chloride and hydrogen peroxide. These findings may indicate a potentially significant role for deep subseafloor sediment fungi in global methane generation, which has not been unaccounted for in previous estimations. Further elucidation of the mechanisms governing methane production by sediment fungi in the deep biosphere promises to advance our understanding of the Earth’s additional sources of methane that contribute to global climate change.

## Figures and Tables

**Table 1:** Relative expression of genes associated with methane synthesis in *S. commune* 20R-7-F01.

	1 d				3 d				5 d			
	RNA-Seq		qPCR		RNA-Seq		qPCR		RNA-Seq		qPCR	
	15 MPa	35 MPa	15 MPa	35 MPa	15 MPa	35 MPa	15 MPa	35 MPa	15 MPa	35 MPa	15 MPa	35 MPa
*mct1*	1.16	1.69	1.21 ± 0.03	1.85 ± 0.11	2.56	2.99	2.82 ± 0.17	3.25 ± 0.21	3.49	5.15	3.17 ± 0.15	5.36 ± 0.19
*mct2*	0.42	0.73	0.37 ± 0.02	0.61 ± 0.05	0.52	0.85	0.44 ± 0.05	0.65 ± 0.07	−0.09	−0.08	−0.1 ± 0.03	−1.7 ± 0.03
*dh1*	0.91	0.62	0.85 ± 0.04	0.88 ± 0.06	0.82	−0.31	0.73 ± 0.02	−0.43 ± 0.04	0.81	−0.73	0.66 ± 0.05	−0.88 ± 0.05
*dh2*	0.21	0.89	0.27 ± 0.03	0.73 ± 0.09	0.85	0.55	0.91 ± 0.06	0.77 ± 0.08	1.48	0.87	1.12 ± 0.11	0.69 ± 0.07
*dh3*	0.68	1.07	0.93 ± 0.06	1.21 ± 0.16	1.48	2.16	1.69 ± 0.11	2.49 ± 0.19	2.11	3.21	1.95 ± 0.13	3.11 ± 0.11
*dh4*	0.56	2.05	0.71 ± 0.03	2.31 ± 0.14	−0.26	−0.24	−0.43 ± 0.03	−0.22 ± 0.01	0.65	−0.23	0.51 ± 0.04	−0.28 ± 0.01
*dh5*	−1.72	−2.04	−1.66 ± 0.04	−2.15 ± 0.15	−1.39	−1.36	−1.31 ± 0.12	−1.46 ± 0.06	−0.13	−0.18	−0.22 ± 0.01	−0.32 ± 0.03
*ms*	1.04	1.12	1.12 ± 0.02	1.25 ± 0.08	1.42	1.63	1.66 ± 0.07	1.77 ± 0.02	1.48	1.51	1.31 ± 0.08	1.63 ± 0.07

**Table 2:** Oxidative levels of *S. commune* 20R-7-F01 under high hydrostatic pressure.

	**ROS (AU/g)**					**H_2_O_2_ (μmol/g)**			
	**0.1 MPa**		**15 MPa**	**35 MPa**		**0.1 MPa**	**15 MPa**		**35 MPa**
1 d	0.37 ± 0.09		1.14 ± 0.22	2.84 ± 0.23		0.31 ± 0.03	0.99 ± 0.12		2.33 ± 0.21
3 d	0.85 ± 0.13		4.46 ± 0.31	6.37 ± 0.21		0.76 ± 0.09	3.66 ± 0.35		4.34 ± 0.37
5 d	0.89 ± 0.11		6.27 ± 0.58	9.19 ± 0.77		0.77 ± 0.08	4.71 ± 0.29		6.55 ± 0.49
	**SOD (U/g)**			**CAT (U/g)**			**POD (U/g)**		
	**0.1 MPa**	**15 MPa**	**35 MPa**	**0.1 MPa**	**15 MPa**	**35MPa**	**0.1 MPa**	**15 MPa**	**35 MPa**
1 d	7.65 ± 0.63	20.94 ± 1.66	26.89 ± 1.53	9.40 ± 0.55	14.87 ± 1.13	23.28 ± 1.51	5.95 ± 0.62	9.18 ± 0.92	35.25 ± 2.53
3 d	14.27 ± 1.37	26.57 ± 1.38	37.78 ± 1.22	12.73 ± 1.06	23.21 ± 1.47	37.25 ± 1.73	15.95 ± 1.33	24.62 ± 1.31	94.56 ± 3.22
5 d	15.92 ± 1.03	28.45 ± 1.54	43.22 ± 1.71	14.54 ± 1.25	25.51 ± 1.64	40.03 ± 2.21	16.35 ± 1.03	25.50 ± 1.52	97.29 ± 3.71

**Table 3:** Effects of varying oxidative stress conditions on methane production in *S. commune* 20R-7-F01.

	CK	CdCl_2_			CdCl_2_ + BHT			H_2_O_2_			H_2_O_2_ + BHT		
		0.75	1.5	3	0.75	1.5	3	0.75	1.5	3	0.75	1.5	3
1 d	0.56 ± 0.04	0.64 ± 0.03	0.91 ± 0.05	1.07 ± 0.02	0.60 ± 0.01	0.81 ± 0.03	0.97 ± 0.01	0.76 ± 0.02	1.03 ± 0.04	1.26 ± 0.06	0.68 ± 0.01	0.87 ± 0.03	1.08 ± 0.01
3 d	0.81 ± 0.03	0.90 ± 0.06	1.54 ± 0.07	1.82 ± 0.05	0.83 ± 0.04	1.40 ± 0.06	1.65 ± 0.07	1.05 ± 0.05	1.74 ± 0.05	2.48 ± 0.09	0.89 ± 0.02	1.52 ± 0.04	2.07 ± 0.04
5 d	0.86 ± 0.06	1.25 ± 0.08	1.69 ± 0.06	2.10 ± 0.09	1.15 ± 0.05	1.53 ± 0.07	1.87 ± 0.03	1.50 ± 0.03	2.09 ± 0.11	2.63 ± 0.12	1.26 ± 0.06	1.85 ± 0.08	2.23 ± 0.05

## Data Availability

No data were used for the research described in the article.

## Supplementary Materials

The following supporting information can be downloaded at: https://www.mdpi.com/article/10.3390/microorganisms12112160/s1.
