# Supplementary material for: Fungal Methane Production Under High Hydrostatic Pressure in Deep Subseafloor Sediments
Source: Microorganisms. 2024 Oct 26;12(11):2160. doi: 10.3390/microorganisms12112160 (PMC11596643; doi:10.3390/microorganisms12112160)
Supplement: Supplementary file 1 [file microorganisms-12-02160-s001.zip › microorganisms-3175360-supplementary.pdf]

Supplementary material

Fungal Methane Production Under High Hydrostatic Pressure in Deep Subseafloor Sediments

Mengshi Zhao[a], Dongxu Li [a], Jie Liu[b], Jiasong Fang[bc]*, Changhong Liu [a,]*

[a]State Key Laboratory of Pharmaceutical Biotechnology, Nanjing University, Nanjing 210023, China

[b]Shanghai Engineering Research Center of Hadal Science and Technology, College of Marine Sciences, Shanghai Ocean University, Shanghai 201306, PR China

[c]Laboratory for Marine Biology and Biotechnology, Qingdao National Laboratory for Marine Science and Technology, Qingdao 266237, China

*Correspondence:
Jiasong Fang
jsfang@shou.edu.cn
Changhong Liu
chliu@nju.edu.cn

Table S1. Transcriptome quality control analysis

Sample_ID	Raw_reads	Raw_bases	Clean_reads	Clean_Bases	Q20%	Q30%
d1_01M1	61225688	9245078888	59924560	8923556763	98.59	95.67
d1_01M2	57660352	8706713152	57093920	8492669667	98.63	95.76
d1_01M3	78692462	11882561762	77966734	11574682555	98.72	96.03
d1_15M1	58292626	8802186526	57372462	8546726611	98.41	95.32
d1_15M2	57596182	8697023482	57148878	8523100243	98.38	95.23
d1_15M3	65583050	9903040550	64891218	9628476854	98.4	95.33
d1_35M1	62930370	9502485870	62248622	9273294662	98.38	95.28
d1_35M2	50448150	7617670650	49998534	7475352356	98.3	95.02
d1_35M3	49151190	7421829690	48754856	7270718571	98.46	95.42
d3_01M1	50533726	7630592626	48120520	7154045635	98.48	95.36
d3_01M2	57806778	8728823478	55059002	8197060519	98.6	95.7
d3_01M3	57385564	8665220164	56966126	8507516649	98.71	95.98
d3_15M1	61646368	9308601568	61022202	9085820646	98.47	95.54
d3_15M2	65798042	9935504342	65238428	9738132671	98.39	95.29
d3_15M3	56952974	8599899074	56250886	8366846120	98.46	95.49
d3_35M1	61443184	9277920784	60730442	9069017317	98.4	95.34
d3_35M2	47487154	7170560254	46292670	6938107353	98.36	95.23
d3_35M3	47088886	7110421786	46439164	6913881206	98.39	95.33
d5_01M1	57971786	8753739686	57444428	8573357523	98.74	96.03
d5_01M2	46008078	6947219778	45531714	6851055859	98.58	95.59
d5_01M3	52839130	7978708630	52454630	7886413231	98.78	96.16
d5_15M1	61865750	9341728250	61253746	9122127602	98.42	95.33
d5_15M2	50901704	7686157304	50333004	7484406434	98.51	95.6
d5_15M3	49558750	7483371250	49052310	7267314405	98.51	95.66
d5_35M1	44510344	6721061944	43941122	6551168123	98.5	95.58
d5_35M2	43622492	6586996292	43113272	6448029277	98.56	95.7
d5_35M3	49276522	7440754822	48618848	7253078402	98.52	95.66

Note: d1_01, d3_01, d5_01 respectively represent strains grown at 0.1MPa for one, three and five days ; d1_15, d3_15, d5_15 respectively represent strains grown at 15 MPa for one, three and five days;d1_35, d3_35, d5_35 respectively represent strains grown at 35 MPa for one, three and five days;Q20: The percentage of bases in clean reads with a Phred value >20; Q30: The percentage of bases in clean reads with a Phred value >30.

Table S2. qRT-PCR primers

Primer ID	Sequence (5'-3')
mct1-F	ATTCCTGGGATATTGCATGG
mct1-R	GTCGAATATGCGAGTTAGCTC
mct2-F	CTCCGGGACCAGAGACAGTT
mct2-R	AGCCAGGGGTGATGTTCTTG
dh1-F	GAATCTCGCCGCTACCAACT
dh1-R	GACGAGAACGCTGATCCAGTA
dh2-F	GTTGGCCGGACTCAACACTA
dh2-R	GTTCACCAACAAGCGCACAT
dh3-F	TCACTCCCCAGCCCAACC
dh3-R	GCTTGTCCGTCTCGCCG
dh4-F	GGCGGCACCAAATCGAATAC
dh4-R	TTGTACCAGTTGAGCGGACC
dh5-F	TTCCAGAAGGGCGGTCTGA
dh5-R	GGCTTCGTGACTTTGAGGGT
ms-F	CATCCCCAGCGGTGATTTCT
ms-R	GGCCCATCGCAAAGTAGACA
act-F	GTCCGCCCTCGAGAAGAGTTA
act-R	TTGTACGTCGTCTCGTGGATA

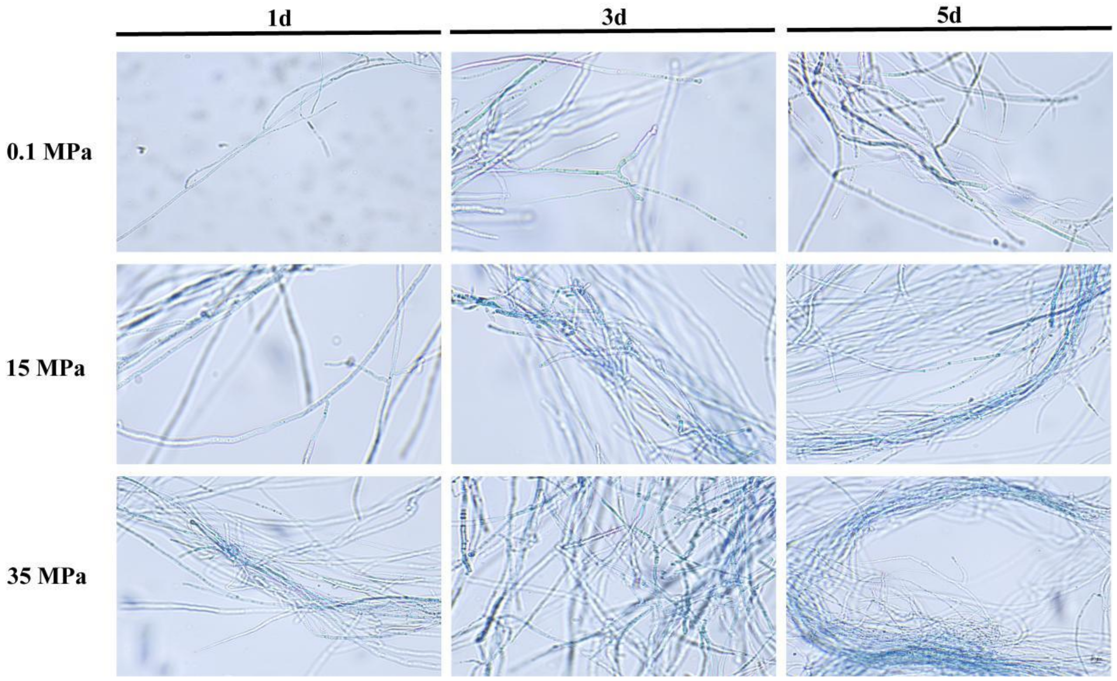

Figure S1. *S. commune* 20R-7-F01 cell activity under high hydrostatic pressure

Table S3. Intracellular O^{2-} and OH· levels of *S. commune* 20R-7-F01 under different stress conditions

	O^{2-} (μmol/g)			OH· (μmol/g)		
	**0.1MPa**	**15MPa**	**35MPa**	**0.1MPa**	**15MPa**	**35MPa**
1d	0.21 ± 0.01	0.24 ± 0.02	0.34 ± 0.03	0.13±0.01	0.19±0.02	0.13±0.01
3d	0.33 ± 0.05	0.26 ± 0.01	0.37 ± 0.01	0.16±0.02	0.16±0.03	0.14±0.03
5d	0.25 ± 0.01	0.31 ± 0.01	0.35 ± 0.02	0.17±0.03	0.21±0.02	0.25±0.04

Table S4. Intracellular ROS levels of *S. commune* 20R-7-F01 under different stress conditions

	$CdCl_2$			H_2O_2		
	**0.75mM**	**1.5mM**	**3mM**	**0.75mM**	**1.5mM**	**3mM**
1d	1.13 ± 0.21	2.31 ± 0.52	2.92 ± 0.55	1.81 ± 0.31	2.54 ± 0.46	3.17 ± 0.65
3d	2.23 ± 0.18	4.78 ± 0.27	5.42 ± 0.46	2.74 ± 0.25	5.23 ± 0.60	7.12 ± 0.33
5d	3.99 ± 0.16	6.82 ± 0.37	8.15 ± 0.32	4.95 ± 0.45	8.65 ± 0.61	10.29 ± 0.47

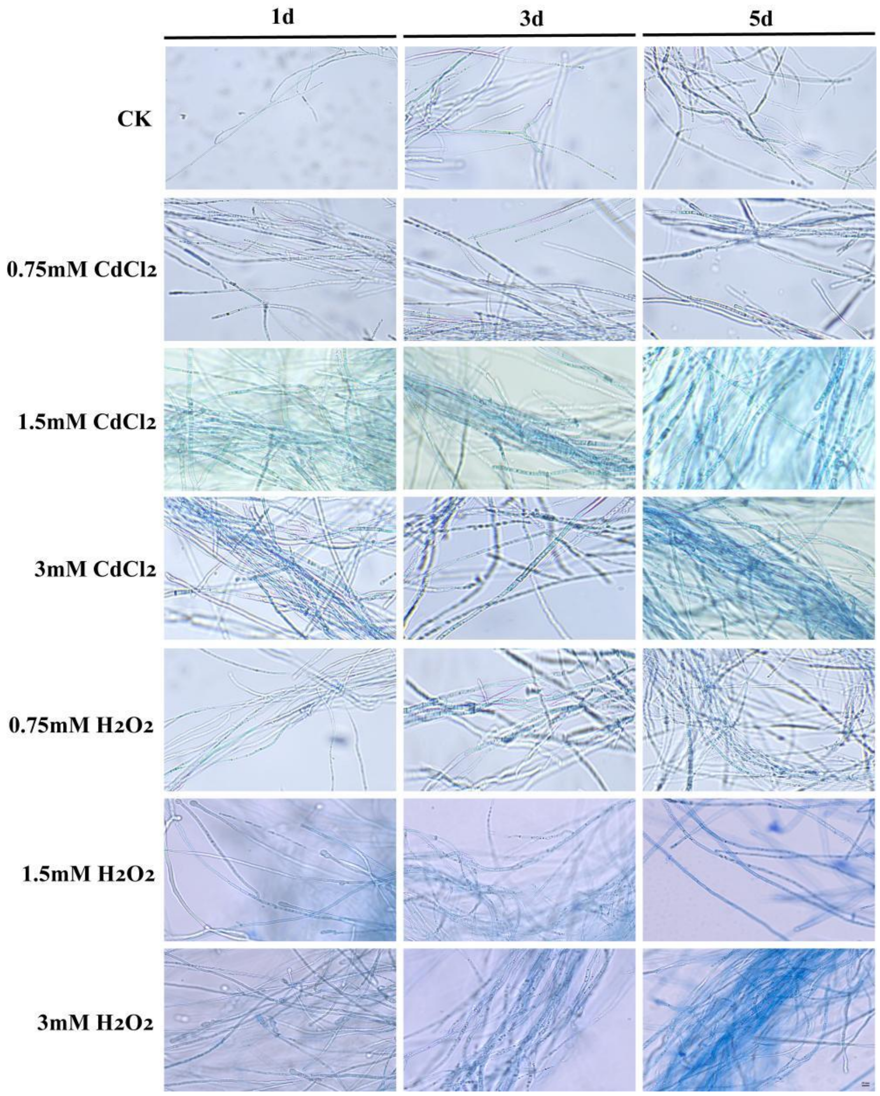

Figure S2. *S. commune* 20R-7-F01 cell activity under different stress conditions

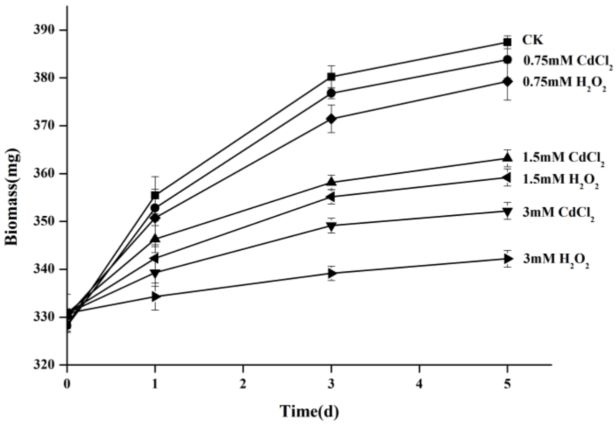

Figure S3. Alterations in the biomass of *S. commune* 20R-7-F01 under varying stress conditions.
